# Health literacy among participants from neighbourhoods with different socio-economic statuses in the southern region of Hungary: a pilot study

**DOI:** 10.1186/s12889-020-08959-0

**Published:** 2020-08-17

**Authors:** Henrietta Bánfai-Csonka, Bálint Bánfai, Sára Jeges, Brigitta Gyebnár, József Betlehem

**Affiliations:** 1 Faculty of Health Sciences, Doctoral School of Health Sciences, University of Pécs, Pécs Vörösmarty st. 4, Pécs, 7622 Hungary; 2 Faculty of Health Sciences, Institute of Emergency Care and Pedagogy of Health, University of Pécs, Pécs Vörösmarty st. 4, Pécs, 7622 Hungary; 3 Department of Children Affairs, Women’s Policy and Equal Opportunities, Ministry of State for Family and Youth Affairs, Ministry of Human Capacities, Szalay st.10-14, Budapest, 1055 Hungary

**Keywords:** Health literacy, Emergency care, Health literacy sub-index, Low-income populations

## Abstract

**Background:**

Health literacy (HL) has a deep impact on people’s decisions about their health and health care system. Measurement and improvement of HL level is essential to develop an appropriate health care system. The aim of the study was to (1) conduct a pilot study among the population of Baranya County in Hungary with different socio-economic statuses, (2) evaluate the HL level and (3) found the correlations between socio-economic data, emergency departments’ visits, medical history and HL.

**Methods:**

In a cross-sectional study conducted in 2019 with 186 participants, socio-economic status, health status, HL level and knowledge about the triage system were measured. The questionnaire included questions on socio-economic status, previous chronic diseases, and satisfaction with the emergency care system as well as the standardised European Health Literacy Survey Questionnaire (HLS-EU-Q47). Descriptive statistical analysis (mean, SD, mode) and mathematical statistical analysis (ANOVA, chi^2^ test, Pearson Correlations, Two sample t-test) were applied. SPSS 24.0 statistical software was used to analyse the data. Relationships were considered significant at the *p* < 0.05 level.

**Results:**

One hundred and eighty-six people were involved in the research, but 45 of them were excluded (*N* = 141). The participation rate was 75.8%. There were significant differences in HL levels by gender and educational level (*p* = 0.017), health education (*p* = 0.032) and presence of children in the household (*p* = 0.049). Educational level (*p* = 0.002) and type of settlement (*p* = 0.01) had strong impacts on economic status. We found that 46.1% of the participants had limited comprehensive HL (cHL) level. This proportion was slightly lower for the disease prevention sub-index (33.3%). The average cHL index score was 34.8 ± 8.7 points, the average health care sub-index score was 34.6 ± 9.7 points, the average disease prevention sub-index score was 35.8 ± 9.9 points, and the average health promotion sub-index score was 34.2 ± 9.4 points. 46.1% of the examined population in Hungary had limited HL level.

**Conclusions:**

Socio-economic status has a strong influence on HL level. It is not enough to improve awareness but we need to improve knowledge and cooperation with the doctors and health care system.

## Background

Decisions made by patients have profound impacts on the health care system, diagnostic and therapeutic decisions, and the disease prevention procedures [1].

Health literacy (HL) is the skill of accessing, understanding and using health information and making decisions about one’s health and health care [1]. A patient with an adequate HL level has the appropriate information to make adequate decisions about his/her own health, their dependents’ health and community health [2]. There are many instruments to measure HL level, the most common of which are the Short Test of Functional Health Literacy (S-TOFHLA), Test of Functional Health Literacy (TOFHLA) [3], Health Literacy Questionnaire (HLQ), Newest Vital Sign (NVS) and Rapid Estimate of Adult Literacy in Medicine Health Literacy Test (REALM) [3]. In Europe, the European Health Literacy Survey Questionnaire (HLS-EU-Q47) was used for a continental survey that included 8 European countries [4].

The incidence of limited HL level is more common than expected. In Europe, 1 in 10 people has insufficient HL level, and one in two has limited HL level [4]. In Portugal, almost three of four people [5] has limited HL level, and every second person in Hungary has limited HL level [6]. Low HL level can cause a higher number of return emergency department (ED) visits [7] and higher mortality (12.8%) due to cardiovascular disease (CVD) in a year of hospitalisation [8]; however, with adequate education, these risks can be reduced [9]. For people with more chronic diseases [10, 11] and for the ageing population [10, 12, 13], it is more difficult to find and understand information. Inadequate and marginal HL level is associated with socioeconomic and self-related health status. People living in rural environment (OR = 2.25) and having poor income (OR = 1.59) were significantly correlated with low HL level which cause more frequent hospital treatments and family doctors’ visits [13]. Low HL level are more frequent in low-income populations [14, 15]. However, some studies have shown no correlations between age [11], socio-economic status [11] and HL. Low HL can also be associated with an unhealthy lifestyle, physical inactivity [16, 17], an unhealthy diet [17], smoking [17] and obesity [16, 17]. For patients with CVD, their HL level are associated with their health behaviours and health status [18].

HL can be identified as a key point in public health [19], but the public and government have been involved in only some recent research on this topic [20]. Schools may be the best place to start to develop HL [21], but adults can also be involved in HL programmes [22].

Why is it difficult to make the correct decisions about our health? First, in the age of the internet and social media, we cannot stop or control the misleading information. There are many forums with fake news that can provide inadequate information to the public [23]. If health care managers would like people to use the correct websites with correct information, they should make these sites modern and user friendly with simple designs [24]. Public awareness and HL can also be increased with the help of social media related to CVD and emergency cases [25]. HL also affects patient-physician communication [3]. This gap can be the reason why physicians and patients think in different ways [26]. Hospital materials are also written in complex form; for better communication, these materials need to be improved [3]. Patients need more attention and compassion from providers [27].

To the best of our knowledge, a previous study in Hungary that compared HL level based on medical and socio-economic status, especially in populations with low socio-economic status has not been conducted so far. Therefore, the aim of this pilot study was to investigate the HL level among low-income populations in the southern part of Hungary in Barany County. We wanted to measure the indicators of health literacy along which we can create a “health literacy program”. We measured HL level and the level of knowledge about triage system and the success with EDs. We also assessed participants’ vital parameters and medical histories regarding previous chronic diseases. We were looking for relationships between HL level and socio-economic data, medication habits and knowledge.

## Methods

A cross sectional study was conducted to investigate the HL level among people with low socio-economic status and to found correlations between visiting and satisfaction with ED units, knowledge about the triage system and patients’ medical habits.

### Study population

Participants were recruited by the Health Development Office in Sellye between April and May 2019. For convenience sampling the respondents were from two villages and one town in Baranya county. One hundred and eighty-six people were involved in the study. All of them were above 18 years. Those who suffered from mental health disease, who are in social care or who did not fit the whole questionnaire were excluded. All participants received an informative document about the survey protocol and data collection, including about the collection of anamnestic data, vital parameters, knowledge about the health care system, especially knowledge about emergency care and HL level.

### Data collection

Data collection was conducted with the following test battery, consists of two main parts. At the beginning the socio-economic part consisted of questions on age, gender, educational level, health educational level, settlement status, marital status, and household income. Regarding the participants’ health conditions, we measured their blood pressure, pulse, oxygen saturation, body weight, and body height and asked questions to record medical history and collect information on their chronic diseases and medication habits. The normal BMI value was considered between 18.5 and 24.99 [28]. In reference to knowledge levels, there were some questions about the triage system.

A Likert scale was used to measure satisfaction with the emergency care system, workers and information system. Visits to family doctors on duty were excluded from this part of the research.

At the second part of the questionnaire the HLS-EU-Q47 questionnaire was used to assess HL level. The HLS-EU-Q47 has 4 indices, i.e., the comprehensive HL index (cHL) and 3 specific dimension sub-indices: the health care (HC), disease prevention (DP) and health promotion (HP) sub-indices. Four categories are used to characterise respondents’ HL level: inadequate, problematic, sufficient and excellent [2, 4]. This questionnaire was previously validated in Hungary [6] and showed good internal reliability (Cronbach’s alpha for the cHL index: 0.97; Cronbach’s alpha for the HC sub-index: 0.92; Cronbach’s alpha for the DP sub-index: 0.92; and Cronbach’s alpha for the HP sub-index: 0.93) [6].

First, the validity of the sub-indexes was tested because the answers of each participant could be considered only if the participant provided fewer than 4 “I don’t know” answers. Only 1 “I don’t know” answer was allowed for questions 1–16 (HC sub-index) and for questions 17–31 (DP sub-index), and 2 “I don’t know” answers were allowed for questions 32–47 (HP sub-index). Thirty-four percent of the participants had one “I don’t know” answer for the HC sub-index, 22.7% had one “I don’t know” answer for the DP sub-index, and 34.8% had one “I don’t know” answer for the HP sub-index.

The coherence between the questions in the cHL index and sub-indexes was high, and the Cronbach’s alpha values were as follows: cHL index: 0.976, HC: 0.933, DP: 0.937 and HP: 0.919.

Second, the cHL index and sub-index descriptive values were measured. The participants’ HL level between 0 and 25 points were considered inadequate, between 26 and 33 points were considered problematic, between 34 and 42 points were considered sufficient and between 43 and 50 points were considered excellent. Participants in the inadequate and problematic groups were considered to have limited HL level. The statistical analysis of this part of the survey was performed as described in previous studies [2, 6].

All participants had to complete the questionnaire with an interviewer, who was a heath care worker and knew the rules for completing the questionnaire. The interviewer were in a separated room with the participants, they could not hear each other during the answering process. First the anthropometric dates were measured, after that they answered the questions.

### Statistical analysis

Descriptive statistical analysis (mean, SD, percentage, mode) was used to describe the sample. Mathematical statistical analysis (ANOVA, chi^2^ test, Pearson correlations, Two sample t-test) was used to determine the relationships between the nominal variables (e.g., gender, educational level) and continuous variables (e.g., HL level). One-way ANOVA and t-test was applied to test the association between HL level, knowledge (knowledge about the triage system), satisfaction with the ED visits and demographic characteristics (gender, education level, type of settlement, health education economic status, marital status, health education, children are household). Pearson correlation was applied to test HL level (e.g. HL level vs. age). SPSS 24.0 statistical software was used to analyse the data. Relationships were considered significant at the *p* < 0.05 level.

## Results

### Socio-economic results

One hundred and eighty-six people were involved in the research, but 45 of them were excluded (*N* = 141) because they did not have time to complete the whole questionnaire or they did not want to answer the socio-demographic questions. The participation rate was 75.8%. The description of socio-economic data can be found in Table 1.

**Table 1:** Description of the socio-economic data

Characteristics	Male	Female	Sample
	41 (29.1%)	100 (70.9%)	141 (100%)
Age (year)	46.8 ± 16.52	45.59 ± 12.75	45.94 ± 13.9
Educational level			
Less than primary school	0	1 (1%)	1 (0.7%)
Primary school	10 (24.4%)	31 (31%)	41 (21.9%)
Vocational school (did not graduate)	10 (24.4%)	4 (4%)	14 (9.9%)
Vocational school (graduated)	6 (14.6%)	24 (24%)	30 (21.3%)
Grammar school	6 (14.6%)	12 (12%)	18 (12.8%)
University at the BSc level	5 (12.2%)	20 (20%)	25 (17.7%)
University at the MSc level	4 (9.8%)	8 (8%)	12 (8.5%)
Health education			
Yes	4 (9.8%)	26 (26%)	30 (21.3%)
No	37 (90.2%)	74 (74%)	111 (78.7%)
Type of settlement			
Village	16 (39%)	53 (53%)	69 (49%)
Town	18 (43.9%)	29 (29%)	47 (33.3%)
City	7 (17.1%)	18 (18%)	25 (17.7%)
Economic status (average)			
Below average	18 (43.9%)	47 (47%)	65 (46.1%)
Average	17 (41.5%)	47 (47%)	64 (45.4%)
Above average	6 (14.6%)	6 (6%)	12 (8.5%)
Marital status			
Single	10 (24.4%)	25 (25%)	35 (24.8%)
Married	18 (43.9%)	38 (38%)	56 (39.7%)
Living with a partner	11 (26.8%)	23 (23%)	34 (24.1%)
Divorced	1 (2.4%)	10 (10%)	11 (7.8%)
Widowed	1 (2.4%)	4 (4%)	5 (3.5%)
Child/children in household			
Yes	10 (24.4%)	42 (42%)	52 (36.9%)
No	31 (75.6%)	58 (58%)	89 (63.1%)

There were significant differences in HL level and educational level (*p* = 0.017), health education (*p* = 0.032) and presence of children in the household (*p* = 0.049). Educational level (*p* = 0.002) and type of settlement (*p* = 0.01) had strong impacts on economic status.

### Results from the anamnestic data and vital parameters

The mean body mass index (BMI) of the participants was 26.23, which means that the pilot study population was overweight. A total of 35.4% of the participants had a normal BMI. The mean blood pressure was 125/80 mmHg, which is normal according to the Hungarian Society of Hypertension [29], but some participants had higher values. Those who suffered from hypertension did not take their medications regularly. The oxygen saturation of respondents was in the normal range, but in one case, it was lower (SpO_2_: 88%) but hat participant suffered from chronic obstructive respiratory disease (COPD). Less than half of the participants (46.1%) had to take medications regularly because they had chronic diseases, and 14 (9.9%) had diseases but did not take medications. The prevalence of previous chronic diseases can be found in Table 2. Twenty-four participants (27.6%) did not take their prescribed medications.

**Table 2:** Prevalence of chronic diseases (*n* = 65)

	Yes	No
Hypertension	33 (50.8%)	32 (49.2%)
Diabetes mellitus	7 (10.8%)	58 (89.2%)
Cardiac disease	10 (15.4%)	55 (84.6%)
Arthritis	3 (4.6%)	62 (95.4%)
Pulmonary disease	6 (9.2%)	59 (90.8%)
Psychiatric disease	4 (6.2%)	61 (93.8%)
Other	18 (27.7%)	47 (72.3%)

### Results regarding emergency department visits and knowledge about the triage system

Sixty-nine (48.9%) of the participants had ED visits during the past year. Most of the patients were taken to the ED by the National Ambulance Service with a referral (21.7%) or without a referral (18.8%) from their family doctor (GP). A total of 92.8% of patients thought that it was necessary to go to the ED, but the number of admissions to other hospital wards was low (28.29%). At the end of their ED stays, 92.4% read the outpatient paper, and 27.3% did not inform their GPs about the results of their ED visits.

Regarding the question “Do you know what triage means?”, 65.2% of the participants answered “Yes”, but when they had to select an answer to indicate the definition of triage, only 46.8% of them chose to the correct answer. A total of 86.5% of participants wanted to have more information about the Hungarian health care and emergency health care system. The desired means of receiving information included personal interaction (41.3%), the internet (52.1%), television (33.1%), brochures (22.3%), the radio or other applications (13.2%) and other forums (e.g., school) (8.3%). There was no significant difference in knowledge about the triage system based on the number of ED admissions (*p* = 0.292).

A total of 46.4% of the patients were triaged within 10 min, but 27.5% were triaged only after 30 min; 47.1% of the patients had to wait less than 15 min, and only 10.3% had to wait more than 2 h. In total, 61.2% of the patients had to stay in EDs 1 to 6 h, but 59.7% of them did not know the reason for waiting.

The results of the satisfaction with the ED admission, care, workers and information giving system can be seen in Table 3.

**Table 3:** Satisfaction with ED visits (*n* = 69)

	1 Not at all satisfied	2 Not satisfied	3 Neither satisfied nor dissatisfied	4 Satisfied	5 Very satisfied	6 Have not answered	Mean points
Administrators’ work	9 (13%)	7 (10.1%)	8 (11.6%)	24 (34.8%)	21 (30.4%)	–	3.59
Paramedics’ and nurses’ work	2 (2.9%)	6 (8.8%)	10 (14.7%)	21 (30.9%)	29 (42.6%)	–	3.97
Doctors’ work	3 (4.3%)	5 (7.2%)	14 (20.3%)	18 (26.1%)	29 (42%)	–	3.94
Medical orderlies’ work	5 (7.2%)	3 (4.3%)	13 (18.8%)	22 (31.9%)	23 (33.3%)	3 (4.3%)	3.67
Radiographers’ work	2 (2.9%)	5 (7.2%)	5 (7.2%)	21 (30.4%)	25 (36.2%)	11 (15.9%)	3.42
Information given about results	8 (11.6%)	3 (4.3%)	14 (20.3%)	20 (29%)	24 (34.8%)	–	3.71
Information given about current status	9 (13%)	4 (5.8%)	13 (18.8%)	24 (34.8%)	19 (27.5%)	–	3.58
Information given about the care process	9 (13%)	9 (13%)	17 (24.6%)	17 (24.6%)	17 (24.6%)	–	3.35
Information given about the whole procedure	14 (20.3%)	4 (5.8%)	12 (17.4%)	21 (30.4%)	18 (26.1%)	–	3.36
Cleanliness of the rooms	7 (10.1%)	6 (8.7%)	13 (18.8%)	20 (29%)	23 (33%)	–	3.67

In the examination of differences of satisfaction levels by education level, there were significant differences only for the cleanliness of the rooms (*p* = 0.046), and in the examination by gender, there was a significant difference in the cleanliness of the rooms (*p* = 0.002) as well.

### Health literacy results

The average cHL index score was 34.8 ± 8.7 points, the average HC sub-index score was 34.6 ± 9.7 points, the average DP sub-index score was 35.8 ± 9.9 points, and the average HP sub-index score was 34.2 points ± 9.4 points. The HL level categories in the different indexes can be seen in Table 4.

**Table 4:** HL levels in the different indexes

	Inadequate	Problematic	Sufficient	Excellent
cHL	16.3% (23)	29.8% (42)	32.6% (46)	21.3% (30)
HC	15.6% (22)	32.6% (46)	27.7% (39)	24.1% (34)
DP	14.9% (21)	18.4% (26)	40.5% (57)	26.2% (37)
HP	19.9% (28)	29.7% (42)	29.1% (41)	21.3% (30)

According to Table 4, 46.1% of the examined population in Hungary had limited HL level; 48.2, 33.3 and 49.6% of the participants had limited HL levels as measured by the HC, DP, and HP sub-indexes, respectively. This finding indicates that close to half of the participants had limited HL levels, except for on the DP sub-index.

Table 5 shows the relationship between HL indices and the socio-economic characteristics of the sample.

**Table 5:** Relationships (level of significance) between the HL index scores, sub-index scores and socio-demographic characteristics (binary analysis)

		cHL	HC	DP	HP
Age (years)	p	0.549	0.978	0.490	0.350
	r^a^	−0.051	−0.002	−0,058	−0.080
Gender	p^b^	0.393	0.031*	0.499	0.738
Female	mean	34.6	33.8	35.5	34.5
Male	mean	36.0	37.3	36.8	33.9
Educational level	p^c^	0.018*	0.253	< 0.001*	0.107
primary school	mean	31.2	31.8	30.4	31.5
secondary school	mean	36.7	36.5	38.1	35.5
university degree	mean	36.4	35.2	38.3	35.7
Health education	p^b^	< 0.001*	< 0.001*	< 0.001*	< 0.001*
yes	mean	39.9	39.5	41.7	38.5
no	mean	33.7	33.5	34.3	33.2
Type of settlement	p^c^	0.043*	0.350	0.003*	0.111
village	mean	33.4	33.4	33.5	33.1
town	mean	35.3	35.5	35.9	34.5
county town	mean	38.3	36.3	41.4	37.2
Economic status	p^c^	0.027*	0.025*	0.117	0.022*
below average	mean	33.1	32.4	34.4	32.5
average	mean	36.1	36.7	36.6	35.2
above average	mean	39.3	37.5	40.4	40.1
Marital status	p^c^	0.864	0.891	0.708	0.920
single	mean	34.0	33.8	34.8	33.5
married	mean	35.9	35.8	37.4	34.5
living in relationship	mean	35.2	35.0	35.4	35.3
divorced	mean	33.8	33.6	34.4	33.5
widow	mean	34.8	34.6	34.1	35.9
Children in household	p^b^	0.015*	0.391	0.009*	0.001*
yes	mean	37.3	35.6	38.6	37.7
no	mean	33.6	34.2	34.3	32.4

*significant difference (*p* < 0.05)

^a^Pearson Correlations

^b^Two sample t-test

^c^ANOVA test

There was no significant difference in cHL level by gender (*p* = 0.393). There were significant difference in cHL level by health education (*p* = 0.001), presence of children in the household (*p* = 0.029), educational level (*p* = 0.02), and type of settlement (*p* = 0.36). People living in towns had higher cHL level than those living in villages and cities. Economic status also had a strong impact on cHL level (*p* = 0.035). Examining the relationship between HL level and educational level according to the Scheffe Post Hock Test there was significant difference between the low educational level (primary school) and the high educational level (university degree). According to the residency relationship was found between villagers and those who are living in county towns. People with below-average income regularly had lower HL level than those with above-average income.

Satisfaction and HL level were not correlated significantly (*p* > 0.05); only satisfaction with radiographers showed a significant difference by HL level (*p* = 0.038).

There were also significant differences in cHL level between those who had chronic diseases and were taking medication (*p* = 0.021) or not taking medication (*p* = 0.007).

## Discussion

In this study we measured the HL level among Hungarians from regions with low socio-economic status.

The main achievement of the study was the summary of data from one questionnaire/survey of the demographics, economic and health statuses, knowledge of triage system and satisfaction with the EDs and HL level.

We found that 46.1% of the participants had limited cHL level. This percentage is similar to that of the general Hungarian population [6]. The percentage of participants with limited HL level was slightly lower for the DP sub-index (33.3%). This finding suggests that the study population can understand and act effectively regarding this aspect of health care. They understand the importance of health screenings and know when it is necessary to receive this kind of examination.

One international study [11] found no significant difference in HL level by demographic characteristics. In our research, we found that educational level, health education, type of settlement, children in the household and economic status had strong effects on cHL level. Differences by gender were observed only for the HC and HP sub-indexes, and differences by age were observed only in the DP sub-index. The difference in cHL level by BMI was not significant, but HL increased as BMI decreased. Therefore, obesity can also influence cHL level [16, 17].

Accessing information about health and health care systems via social media is very popular, but the information that social media offer can be unsafe and false [23]. Regardless, most of the participants wanted to obtain information about the health care system and ED via the internet and television. In addition, personal contact is necessary. Schools and outpatient materials can also be good ways to communicate information to the public and patients and to develop HL [21, 22].

### Limitations of the study

We recruited participants from the remaining villages (characterised with low income and isolation) in the region where we intended to collect our data, but we conducted the research in collaboration with the Health Development Office in Sellye, collecting data via their social programmes (“Health Day” and “Parents’ club”). Therefore, we could not exclude people who lived in other settlements in the region or had better social and economic statuses.

We conducted assessments in only one part of Hungary that was selected by the research group, so we cannot ensure that the sample was representative of this region. The modest sample size may also affect the uncertainty of the results.

Data about health and medical histories were self-reported, and we could not determine their reliability. The whole questionnaire was based on self-reported data, so we need to conduct a quantitative part of the study to verify the HL level.

Despite these limitations of the study, we think that the study provides a good basis for a nationwide research.

## Conclusions

The circumstances of the respondents can have an effect on HL level. If we would like to improve HL level in our country, we need to concentrate on regions with low average income as well. It is not enough to improve awareness but we need to improve knowledge and cooperation with the doctors and health care system.

## Data Availability

The dataset supporting the conclusions of this article and the questionnaire is available from the corresponding author on reasonable request.

## Abbreviations

BMI: Body mass index
cHL: Comprehensive health literacy
COPD: Chronic obstructive respiratory disease
CVD: Cardiovascular disease
DP: Disease prevention
ED: Emergency department
GP: General practitioner (family doctor)
HC: Health care
HL: Health literacy
HLQ: Health Literacy Questionnaire
HLS-EU-Q47: European Health Literacy Survey Questionnaire
HP: Health promotion
NVS: Newest Vital Sign
REALM: Rapid Estimate of Adult Literacy in Medicine Health Literacy Test
SD: Standard deviation
SPSS: Statistical package for the Social Sciences
S-TOFHLA: Short Test of Functional Health Literacy
TOFHLA: Test of Functional Health Literacy
